# Integrative Analysis of Bulk RNA-Seq and Single-Cell RNA-Seq Unveils Novel Prognostic Biomarkers in Multiple Myeloma

**DOI:** 10.3390/biom12121855

**Published:** 2022-12-12

**Authors:** Jing Zhao, Xiaoning Wang, Huachao Zhu, Suhua Wei, Hailing Zhang, Le Ma, Pengcheng He

**Affiliations:** Department of Hematology, The First Affiliated Hospital of Xi’an Jiaotong University, Xi’an 710061, China

**Keywords:** multiple myeloma, single-cell sequencing, bulk transcription, cell differentiation trajectory, prognostic signature, tumor immune microenvironment

## Abstract

Molecular heterogeneity has great significance in the disease biology of multiple myeloma (MM). Thus, the analysis combined single-cell RNA-seq (scRNA-seq) and bulk RNA-seq data were performed to investigate the clonal evolution characteristics and to find novel prognostic targets in MM. The scRNA-seq data were analyzed by the Seurat pipeline and Monocle 2 to identify MM cell branches with different differentiation states. Marker genes in each branch were uploaded to the STRING database to construct the Protein-Protein Interaction (PPI) network, followed by the detection of hub genes by Cytoscape software. Using bulk RNA-seq data, Kaplan-Meier (K-M) survival analysis was then carried out to determine prognostic biomarkers in MM. A total of 342 marker genes in two branches with different differentiation states were identified, and the top 20 marker genes with the highest scores in the network calculated by the MCC algorithm were selected as hub genes in MM. Furthermore, K-M survival analysis revealed that higher NDUFB8, COX6C, NDUFA6, USMG5, and COX5B expression correlated closely with a worse prognosis in MM patients. Moreover, ssGSEA and Pearson analyses showed that their expression had a significant negative correlation with the proportion of Tcm (central memory cell) immune cells. Our findings identified NDUFB8, COX6C, NDUFA6, USMG5, and COX5B as novel prognostic biomarkers in MM, and also revealed the significance of genetic heterogeneity during cell differentiation in MM prognosis.

## 1. Introduction

The second most common hematological malignancy, multiple myeloma (MM), is characterized by abnormal accumulations of malignant plasma cells. Compared with other hematological malignancies, MM is characterized by an insidious onset. Clinically, patients first start with a monoclonal gammopathy of undetermined significance (MGUS) having limited myeloma cells, which is a preneoplastic condition. Then the disease progresses to smoldering myeloma (SMM) without end-organ damage [1,2]. Eventually, patients progress to multiple myeloma with clinical manifestations of end-organ dysfunctions [3]. New anti-myeloma therapies including proteasome inhibitors, immunomodulatory agents, and other immunotherapies have substantially improved patient outcomes [4,5]. Ultimately, MM remains incurable due to progression and recurrence. Complex genomic architecture and clonal evolution are believed to contribute to the refractoriness and progression of MM [6]. The heterogeneous outcome of MM is determined by the complex cytogenetic abnormalities with different genomic subclones and biological functions. Thus, there is an increased focus on understanding the clonal evolution of the disease.

A deeper understanding of MM clonal progression has been achieved using next-generation sequencing. Currently, the MM genomic landscape is characterized by initiating events including hyperdiploidy and immunoglobulin translocations, and tumor driver gene mutations known as acquired events [7]. Research has revealed that the premalignant clone is detectable in the preneoplastic period, and that initiating genetic events occurred from the preneoplastic conditions to the progression of the tumor [8]. Deep interrogation of the oncogenesis of MM on a large scale is a challenge due to the heterogeneous cytogenetic abnormalities of MM patients. Branching evolutionary patterns, molecular events including genetic mutations, and copy number changes are critical to MM transition and development [9]. Previous studies demonstrated that fusion genes, copy number, and translocation events impact the prognostic stratification, disease progression, and treatment response of patients [10].

The current risk stratification system of MM relies on cytogenetic markers, or gene expression panels [11]. However, current risk classification based on gene expression abnormalities can only provide a patient cohort-based prediction instead of an individual precise prediction for each patient [12]. Current gene expression prediction panels based on bulk RNA-seq analysis and qPCR are challenging since most samples contain cancer cells, stromal cells, and immune cells that vary greatly from patient to patient. Single-cell sequencing offers an opportunity to reveal the risk of progression or therapeutic resistance at single-cell resolution from different tumor cell populations. The first single-cell genetic analysis of MM was performed in 6 patients with initiating t (11,14), revealing that clone diversity is essential for the progression of MM [13]. Subsequent studies have conducted longitudinal investigations on the genetic heterogeneity of MM progression and recurrence via scRNA-seq [14,15,16]. Meanwhile, based on scRNA-seq, recent studies also have demonstrated the immune changes associated with the multiple myeloma progression [17,18,19].

Single-cell transcriptome analysis has become a crucial approach to studying the complex biological processes of heterogeneous cells. The Monocle2 algorithm is based on the expression matrix of the single-cell transcriptome, which simulates the biological functions of cell populations by unsupervised learning of cells to different branches of the developmental trajectory [20]. In addition, the tSNE algorithm can cluster cells to obtain differential genes in different states and analyze hub genes affecting different branches of differentiation [21]. 

An analysis combining single-cell sequencing and bulk sequencing was performed in this study to decipher cell differentiation-specific gene signatures of multiple myeloma. Our results indicated that during the cell differentiation and disease progression, MM cells can be divided into two specific branches according to gene signatures. Moreover, the clustered malignant plasma cells and the identified genes were validated through bulk transcription. Several of these genes are potential biomarkers associated with the prognosis of MM. In addition, we validate the findings with clinical samples with a quantitative polymerase chain reaction. 

## 2. Materials and Methods

The Materials and Methods should be described with sufficient details to allow others to replicate and build on the published results. Please note that the publication of your manuscript implicates that you must make all materials, data, computer code, and protocols associated with the publication available to readers. Please disclose at the submission stage any restrictions on the availability of materials or information. New methods and protocols should be described in detail, while well-established methods can be briefly described and appropriately cited.

### 2.1. Data Source and Processing

For bulk RNA-seq analysis, the expression and clinical data of 859 MM samples were sourced from the TCGA database (https://portal.gdc.cancer.gov/ (accessed on 18 March 2022)). For scRNA-seq analysis, 15 samples from different MM stages (MGUS = 3, SMM = 4, NDMM = 5, RRMM = 3) in the GSE118900 dataset were extracted from the GEO database [22]. All samples were analyzed on the Illumina HiSeq 2500 platform. scRNA-seq data were analyzed by the Seurat package [23]. First, genes and low-quality cells were filtered using the following criteria: genes detected in ≤ 3 cells, the number of unique genes detected in each cell < 50, and the percentage of reads mapped to the mitochondrial genome ≥ 5%, no cells were filtered out and 597cells were used for the following analysis. The normalization of scRNA-Seq data of high-quality cells was performed and highly variable genes were selected for downstream analysis. Then, we performed principal component analysis (PCA) on highly variable genes and identified the significant principal components (PCs). Different PCs implied different combinations of highly variable genes. PCs with *p* < 0.05 were selected for subsequent analysis. t-Distributed Stochastic Neighbor Embedding (tSNE) analysis was performed for dimension reduction and visualization of gene expression. RunTSNE and TSNEPlot functions in Seurat package were utilized to perform cell clustering. The selected features were used for principal components analysis (PCA). The Find All Markers function was applied to detect marker genes of each cell cluster. The function of marker genes of each cell cluster was analyzed by the clusterProfiler R package. Next, the annotation of cell types in different cell clusters was performed with the SingleR package, and manually determined the clusters based on the marker genes in the CellMarker database [24,25]. Monocle 2 was utilized for pseudo-time analysis to analyze the developmental trajectory of MM cells. For downstream analysis, genes differentially expressed in different differentiation branches were identified as branch marker genes [20]. The function of marker genes of each cell cluster and branch marker genes was analyzed by the clusterProfiler R package.

### 2.2. Correlation Analysis 

To investigate whether bulk RNA-seq data can detect cells in different differentiation branches obtained from scRNA-seq data, we performed Pearson correlation analysis of the expressions of branch marker genes in both TCGA-MM and GSE118900 datasets. In addition, to explore the relationship between different differentiation branches, correlation analysis between metagene expression levels from different differentiation branches was calculated. The metagene expression level was generated by the branch marker gene expression profile [26]. 

### 2.3. Screening Hub Genes Associated with MM Prognosis

The (Protein-Protein Interaction (PPI) network of branch marker genes was developed via the STRING database and Cytoscape software. The Maximal Clique Centrality (MCC) algorithm in the cytoHubba plug-in was used to extract the top 20 branch marker genes (hub genes) in the network [27]. Based on the TCGA, MM patients were divided into groups based on the median expression levels of the hub genes. To identify hub genes associated with prognosis, Kaplan–Meier survival analysis (K–M analysis) and log-rank tests were used (*p* < 0.05). In addition, the expression of prognostic biomarkers was compared between/among groups stratified by MM clinical features by Wilcoxon or Kruskal-Wallis tests.

### 2.4. Relationship between Prognostic Biomarkers and Tumor Microenvironment

Bulk RNA-seq data usually included tumor cells, immune cells, and other various cellular combinations. A single-sample gene set enrichment analysis (ssGSEA) algorithm was performed to quantify the relative immune cell infiltration levels of each sample by GSVA package. The ssGSEA adopted gene biomarkers expressed by immune cell populations to individual TCGA-MM samples. The proportion of immune cells was assessed separately for individual gene expression series; the sum of different immune cell fractions in each sample equaled 1. The abundances of 24 immune cell types were assessed and visualized with bar plots. Then correlations between the expression of prognostic biomarkers and the proportions of the 24 immune cells were evaluated by the Pearson method; |cor| > 0.3 and *p* < 0.05 were considered to be a significant correlation. 

### 2.5. Patients and Samples

The First Affiliated Hospital of the Xi’an Jiaotong University Ethics Committee approved the research protocol. Written informed consent was provided by each patient who participated in the study. The diagnosis criteria used by the IMWG for multiple myeloma were applied to the patients. The clinical stage and risk status of MM patients were determined in accordance with the R-ISS. Healthy donors were used as controls after informed consent was obtained. Bone marrow samples were obtained from patients and healthy controls. The mononuclear cells were separated by lymphocyte separation liquid (Gibco, Carlsbad, CA, USA) within 2 h after the bone marrow samples were harvested. 

### 2.6. Quantitative Real-Time Polymerase Chain Reaction (qRT-PCR)

We extracted total cellular RNA using Trizol reagent (Invitrogen, Carlsbad, CA, USA) according to the manufacturer’s recommendations. Then the total RNA was reverse transcribed to cDNA by HiScript^®^ II Q RT SuperMix for qPCR (Vazyme Biotech, Nanjing, China) with random primers (Promega, Madison WI, USA) for mRNA and lncRNA. The SYBR Green Master Mix reagents (Vazyme Biotech, Nanjing, China) were used in quantitative RT-PCR analysis to amplify and analyze the expression of mRNA in three replicates per sample. All primer sequences are listed in Supplementary Table S1. The relative expression of genes was analyzed using the 2^−ΔΔCT^ method. 

### 2.7. Statistical Analysis

Statistical analyses were conducted by GraphPad Prism 9.0 software (GraphPad Software, San Diego, CA, USA). Based on at least three independent assays, data are presented as mean + standard deviation (SD). Student’s *t*-test and one-way ANOVA test were statistically used to compare two-group and multiple-group. *p* < 0.05 indicated a statistical significance.

## 3. Results

### 3.1. Single-Cell Sequencing Revealed the Cell Distribution of MM and Six Cell Clusters Were Identified in MM

**Figure 1:**
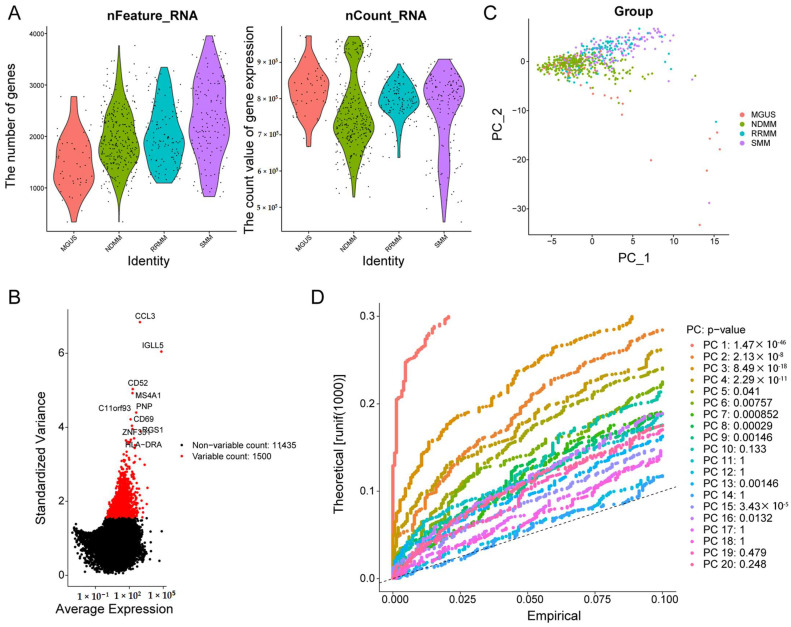
Quality assurance for single cell isolation and sequencing. (**A**) The gene descriptions and total gene numbers for the profiles. (**B**) Diagrams of differentially expressed genes between cells. (**C**) Results of PCA for single-cell sequencing. (**D**) *p* value of each PCA component. PCA: principal component analysis; PC-1: principal component-1; PC-2: principal component-2; MGUS: monoclonal gammopathy of undetermined significance; NDMM: newly diagnosed multiple myeloma; RRMM: relapsed/refractory multiple myeloma; SMM: smoldering multiple myeloma.

**Figure 2:**
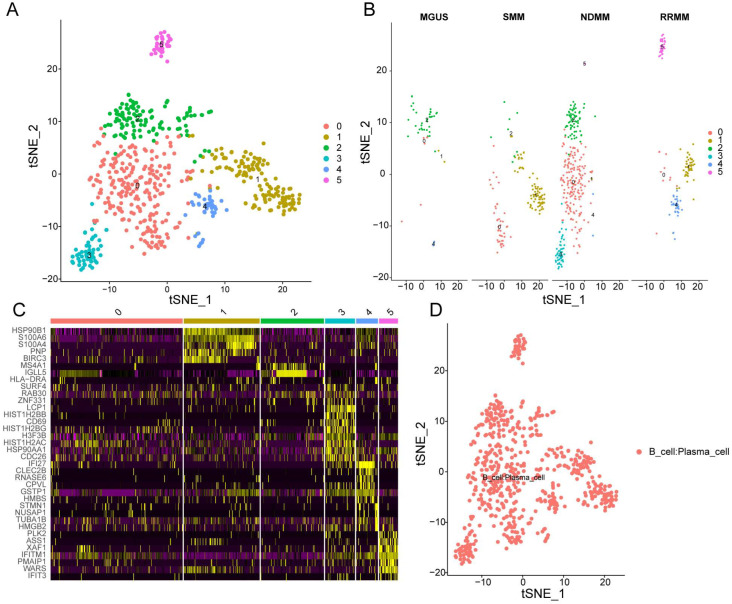
tSNE clustering of single-cell expression profiles. (**A**) tSNE clustering of single-cell transcriptomes colored according to cell−type clusters. (**B**) tSNE clustering of single-cell transcriptomes colored according to different disease stages. (**C**) Heatmap showing the top 5 genes in each cluster. Purple to yellow indicates low to high levels of gene expression. (**D**) Annotation of cell clusters by SingleR and CellMarker database.

After quality control, the results of the number of genes (nFeature) and unique molecular identifiers (nCount) showed that we obtained high-quality cells for downstream analysis (Figure 1A). The top 1500 variable genes were plotted in the scatter diagram, in which CCL3, IGLL5, CD52, MS4A1, C11orf93, PNP, CD69, RGS1, ZNF331, and HLA-DRA with the highest differential gene expression were marked (Figure 1B). According to the PCA results, no clear separation of MM cells at different stages was observed (Figure 1C). Twenty principal components (PCs) were identified, and the top 9 PCs with *p* < 0.05 were selected for analysis (Figure 1D). Thereafter, MM cells were clustered into six groups by the t-SNE algorithm (Figure 2A). Additionally, we observed that MM cells at the MGUS stage were composed of clusters 0, 1, 2, and 4, MM cells at the SMM stage were composed of clusters 0, 1, and 2, and MM cells at the newly diagnosed multiple myeloma (NDMM) stage were composed of clusters 0-5, and MM cells at the relapsed/refractory multiple myeloma (RRMM) stage were composed of clusters 0, 1, 4, and 5 (Figure 2B), indicating the cellular heterogeneity at different MM stages. There were 430 marker genes identified from those six clusters (Table S2), and the expression patterns of the top five markers of each cluster are shown in the heatmap (Figure 2C). By the SingleR and CellMarker databases, all those cell clusters were annotated as plasma cells (Figure 2D). 

### 3.2. Investigation of the Functions of Marker Genes of the Six Cell Clusters 

**Figure 3:**
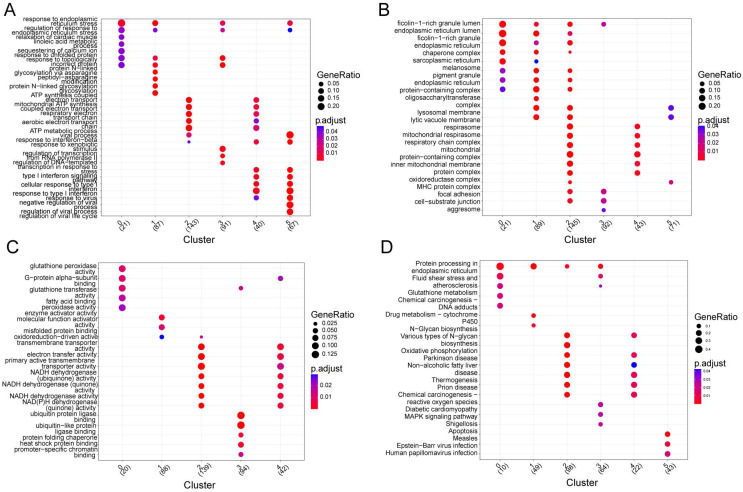
Functional enrichment analysis of genes within sub-clusters. (**A**) Functional enrichment analysis was conducted using the Reactome pathway database; each circular symbol represents an individual pathway. (**B**–**D**) Enriched GO_MF (**B**), GO_CC (**C**), and GO_BP (**D**) pathway annotation of marker genes within different sub-clusters.

Subsequently, annotating each cluster was accomplished by identifying its marker genes (Figure 3 and Tables S3–S6). Those marker genes in different cell clusters had both common and distinct functions. A functional gene set enrichment analysis (GSEA) was performed in order to identify enriched gene ontology (GO) terms across the three domains: biological process (GO-BP), molecular function (GO-MF) and cellular component (GO-CC) [28]. Specifically, marker genes in cluster 0 were significantly enriched in 13 biological processes (BPs), 17 cellular components (CCs), 11 molecular functions (MFs), and 10 KEGG pathways, such as response to endoplasmic reticulum stress, glutathione peroxidase activity, ficolin-1-rich granule lumen, and protein processing in the endoplasmic reticulum. Marker genes in cluster 1 were significantly enriched in 29 BPs, 21 CCs, 3 MFs, and 3 KEGG pathways, such as protein N-linked glycosylation via asparagine, endoplasmic reticulum protein-containing complex, enzyme activator activity, and N-glycan biosynthesis. Marker genes in cluster 2 were significantly enriched in 81 BPs, 84 CCs, 49 MFs, and 20 KEGG pathways, such as ATP synthesis coupled electron transport, respirasome, oxidoreduction-driven active transmembrane transporter activity, and oxidative phosphorylation. Marker genes in cluster 2 were significantly enriched in 81 BPs, 84 CCs, 49 MFs, and 20 KEGG pathways, such as ATP synthesis-coupled electron transport, respirasome, oxidoreduction-driven active transmembrane transporter activity, and oxidative phosphorylation. Marker genes in cluster 3 were significantly enriched in 89 BPs, 4 CCs, 9 MFs, and 6 KEGG pathways, such as the response to unfolded protein, focal adhesion, ubiquitin-protein ligase binding, and MAPK signaling pathways. Marker genes in cluster 4 were significantly enriched in 33 BPs, 11 CCs, 10 MFs, and 6 KEGG pathways, such as the type I interferon signaling pathway, mitochondrial protein-containing complex, oxidoreduction-driven active transmembrane transporter activity, and diabetic cardiomyopathy. Marker genes in cluster 5 were significantly enriched in 122 BPs, 3 CCs, and 3 KEGG pathways, such as regulation of the viral process, MHC protein complex, and measles.

### 3.3. Two Different Differentiation Traces Were Observed in MM Cells

**Figure 4:**
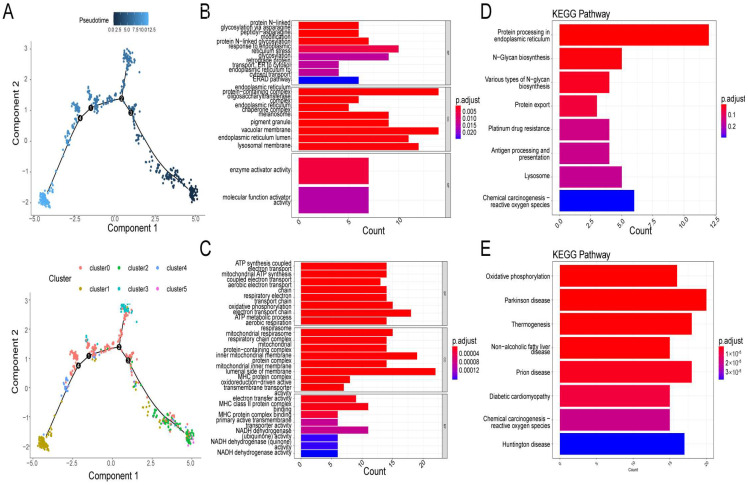
Reconstruction of a cell trajectory by monocle single-cell trajectory analysis. (**A**) Monocle reconstructed two main branches from a single cell trajectory. Branch 1 contains cluster 1 and cluster 4, branch 2 contains cluster 2 and cluster 5. Cells are colored based on state (left), and cluster (right). Cells in different branches have different cellular differentiation characteristics. (**B**–**E**) Gene ontology (GO) enrichment analysis and KEGG pathway analysis for branch−dependent genes. GO enrichment analysis (**B**) and KEGG pathway analysis (**D**) of differentially expressed genes (DEGs) in branch1. GO enrichment analysis (**C**) and KEGG pathway analysis (**E**) of DEGs in branch2.

**Figure 5:**
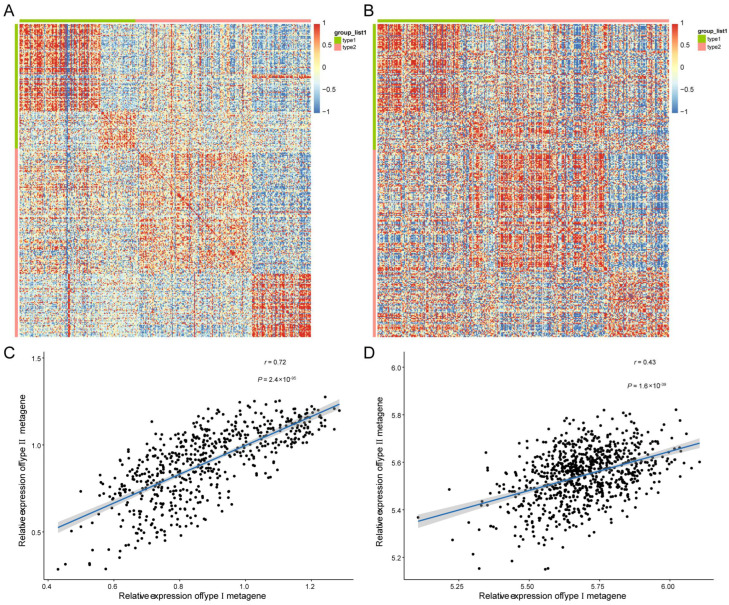
Correlation of bulk and scRNA-seq datasets. (**A**) Heatmap visualization of the DEGs associated with two cell differentiation branches in scRNA-seq datasets. (**B**) Heatmap visualization of the DEGs associated with two cell differentiation branches in TCGA dataset. (**C**) Correlation analysis of metagene scores of Type I and type II marker genes in scRNA-seq dataset. (**D**) Correlation analysis of metagene scores of Type I and type II marker genes in scRNA-seq dataset.

By analyzing the trajectory, the MM cells seemed to start principally from clusters 2 and 5, then moved towards cluster 0 and cluster 3, and finally to cluster 1 and cluster 4 (Figure 4A). After differential expression analysis, 136 and 206 marker genes were detected in branch 1 (cluster 1 and cluster 4) and branch 2 (cluster 2 and cluster 5), respectively (Table S7). Functional analysis showed that marker genes in branches 1 (Type I) and 2 (Type II) were involved in similar GO terms, such as ATP synthesis-coupled electron transport, respirasome, and respiratory electron transport chain (Figure 4B,C). The expression level of Type I and Type II metagene were calculated by the ssGSEA algorithm based on 136 and 206 marker genes’ expression level, respectively. There was a significant enrichment of Type I genes in ER, N-glycan biosynthesis, and protein export (Figure 4D) in KEGG pathways, while Type II genes were mainly enriched in oxidative phosphorylation, Parkinson’s disease, and thermogenesis pathways (Figure 4E). Moreover, by correlation analysis, we found that genes with Type I or Type II labels were highly correlated in bulk and in scRNA-seq (Figure 5A,B), indicating that using those two branch marker genes can also identify the corresponding cells with different differentiation statuses. Moreover, we noted that there was a significant correlation between Type I and Type II metagene relative expression in both scRNA-seq datasets (cor = 0.72, *p* < 0.05, Figure 5C) and bulk RNA-seq datasets (cor = 0.42, *p* < 0.05, Figure 5D), suggesting that different differentiated MM cells may be functionally correlated, which was also consistent with the GO results of marker genes in branches 1 and 2.

### 3.4. Five Candidate Prognostic Biomarkers Were Identified in MM

**Figure 6:**
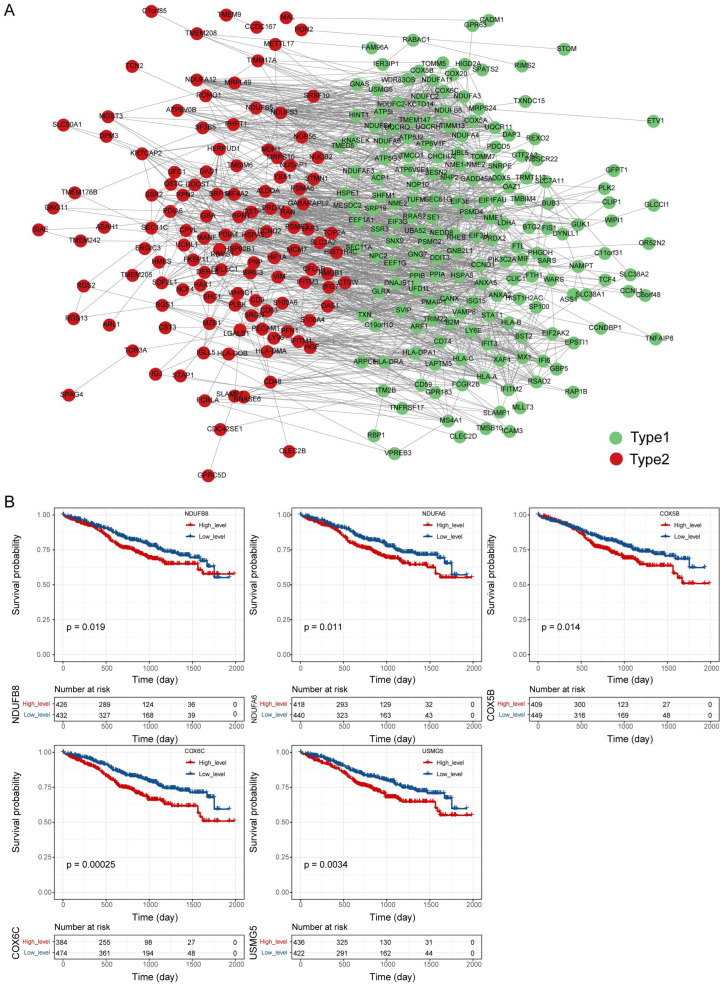
Protein-protein interaction network of DEGs in different cell types and identification of hub genes related to the prognosis of MM (**A**) Protein-protein interaction network of DEGs in Type I and Type II. (**B**) Kaplan–Meier survival curves of MM patients with different expression levels of NDUFB8, NDUFA6, COX5B, COX6C, and USMG5.

**Figure 7:**
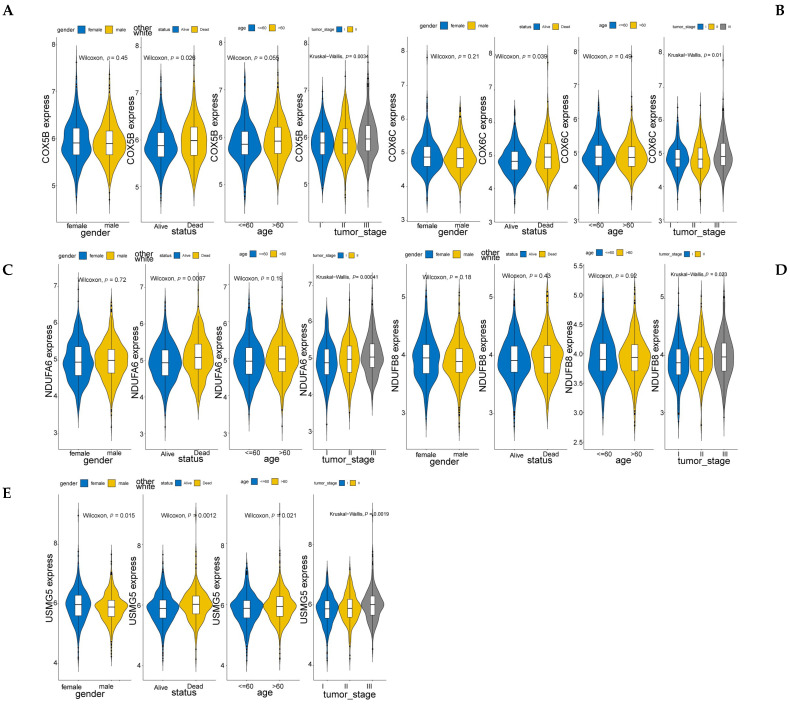
Identification of the correlation between expression of prognostic hub genes and clinical characters. (**A**–**E**) A close correlation was found between NDUFB8, NDUFA6, COX5B, COX6C, and USMG5 expression in MM, the stage of the tumor and the prognosis.

Next, we constructed a PPI network composed of 306 nodes and 1853 edges to show the interaction of Type I and Type II genes (Figure 6A). Then, by the cytoHubba plug-in, we selected the top 20 genes in the network as hub genes in MM (Table S8). On the basis of the median expression level of each hub gene, patients were divided into low- and high-expression groups. K–M analysis showed that the low-expression group with lower expression levels of NDUFB8, NDUFA6, COX5B, COX6C, and USMG5 had significantly better survival than those with higher expression levels (Figure 6B), indicating that NDUFB8, NDUFA6, COX5B, COX6C, and USMG5 may act as candidate prognostic biomarkers in MM. Moreover, the expression of all those five genes increased with the tumor stage (stage III > stage II > stage I) (Figure 7), suggesting their relationship with the progression of MM.

### 3.5. Prognostic Biomarkers Were Related to the MM Tumor Microenvironment 

**Figure 8:**
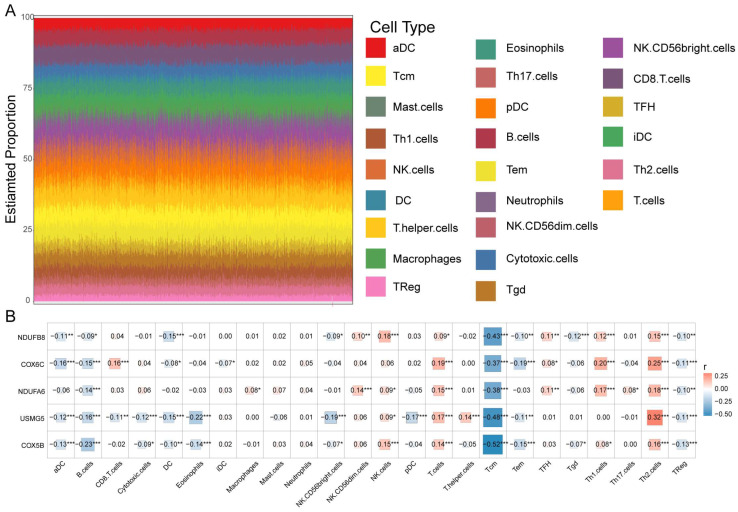
Correlation of immune microenvironment with prognostic hub genes. (**A**) Stacking diagram for immune cell infiltration based on ssGSEA score. (**B**) Correlation between the expression of NDUFB8, NDUFA6, COX5B, COX6C, USMG5, and different immune cell infiltration. * *p* < 0.05, ** p < 0.01, *** p < 0.001.

The tumor immune microenvironment (TIM) is crucial to the pathobiology of MM [29]. Thus, we determined whether prognostic biomarkers are associated with the TIM of MM. First, we analyzed the proportion of 24 immune cells in MM samples (Figure 8A). Then, Pearson’s analysis showed that the expression levels of all five biomarkers were negatively correlated with the proportion of central memory T-cells (Tcm) (cor < −0.3, *p* < 0.05) (Figure 8B), suggesting that the prognostic biomarkers may affect MM by interacting with Tcms.

### 3.6. Verification of Diagnostic Markers

**Figure 9:**
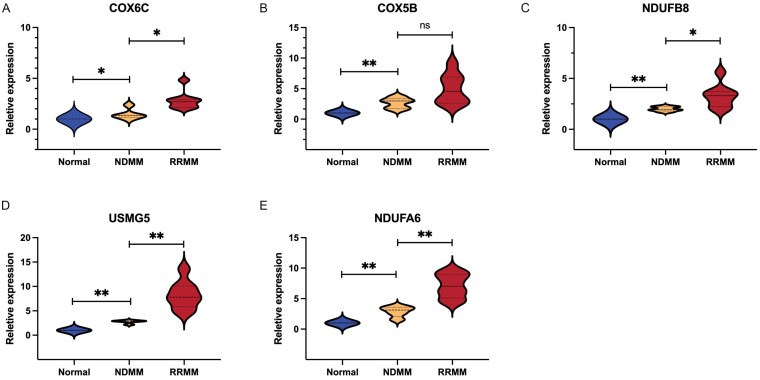
Validation of prognostic hub genes in clinical samples. (**A**–**E**) Relative expression of COX6C, COX5B, NDUFB8, USMG5, and USMG5 in different stages of MM patients.* *p* < 0.05, ** p < 0.01.

The expression levels of NDUFB8, NDUFA6, COX5B, COX6C, and USMG5 were detected by qRT-PCR in 18 bone marrow samples including 5 controls, 5 newly diagnosed MM patients, and 8 refractory /relapsed MM patients. As presented in Figure 9, MM patients showed higher expression of NDUFB8, NDUFA6, COX5B, COX6C, and USMG5 than healthy controls. Additionally, relapsed/refractory myeloma patients expressed significantly higher expression of NDUFB8, NDUFA6, COX6C, and USMG5 than newly diagnosed patients. The result indicated that the NDUFB8, NDUFA6, COX5B, COX6C, and USMG5 genes might be closely related to the disease progression and the evolution of high-risk clones.

## 4. Discussion

Multiple myeloma is the second most common hematological cancer. Due to a lack of early detection and precise stratification, patients suffer from relapse and refractoriness despite recent improvements in therapies. MM is a plasma cell malignancy. It occurs when malignant plasma cells expand clonally within the bone marrow. MM is age-dependent with high complexity and heterogeneity at the molecular level. The genetic landscape of MM has now been well described due to the result of high throughput sequencing technical developments [30]. Some disease-associated recurrent mutations have been identified as markers for risk classification [11]. Nevertheless, intratumoral and interpatient genetic heterogeneity and dynamic clonal evolution are obstacles to the delivery of effective precision medicines to MM patients. Deciphering key features within MM clonal composition and clonal evolution is essential for personalized therapy selection. Single-cell sequencing provides great insight into the evolution and diversity of cancer. It is becoming a valuable tool for dissecting and understanding the essential role of rare cells in tumor progression. 

In the present study, we used scRNA-Seq combined with bulk RNA-Seq to describe the tumor heterogeneity and tumor immune microenvironment of MM. We deciphered the common features of myeloma cells during all stages of the disease and identified the cellular subpopulations. Furthermore, we found two distinct cell differentiation trajectories in the dynamic progression of MM by the scRNA-Seq dataset. Functional analysis indicated that marker genes in the Type I branch were mainly involved in protein processing in the ER, N-glycan biosynthesis, and protein export in KEGG functional pathways, while Type II genes were mainly enriched in oxidative phosphorylation and thermogenesis. Studies have described the pivotal role of endoplasmic reticulum (ER) stress in myeloma pathogenesis. MM cells are extremely sensitive to ER stress-induced cell death. For that reason, drugs including proteasome inhibitors and novel ER stressors that disrupt ER homeostasis have demonstrated remarkable therapeutic effects in MM [31]. On the other hand, dysregulation of energy metabolism and oxidative phosphorylation changes are common features of different tumors. Multiple myeloma cell proliferation depends on both oxidative phosphorylation and glycolysis following a mitochondrial transfer from bone marrow stromal cells [32]. Oxidative phosphorylation was identified as one of the causes mediating therapeutic resistance of B-cell hematological malignancy [33]. The Multiple Myeloma Research Foundation (MMRF) CoMMpass clinical research study indicates the overexpression of genes involved in oxidative phosphorylation in the newly diagnosed multiple myeloma patients enrolled in the dataset [34]. Furthermore, MM patients who expressed high levels of oxidative phosphorylation genes had a worse prognosis [35]. In treated individuals, oxidative phosphorylation activation was observed as a metabolic resistance in MM patients and cell lines, which indicates that the oxidative phosphorylation pathway may be as a preferential target of relapsed/refractory multiple myeloma patients [36]. Our findings that prognostic characteristic genes may influence the prognosis of MM patients by regulating ER-stress-associated cell death and oxidative phosphorylation, are consistent with the above literature. 

In addition, a bulk RNA-Seq analysis based on the scRNA-Seq results identified differentially expressed genes (DEGs) that contribute to the differentiation of MM. To further investigate the further mechanism of DEGs in the differentiation of MM, we analyzed the PPI network of those DEGs. Combined with the K–M analysis, we found the high expression of five genes in MM patients is closely related to tumor progression. NDUFB8 and NDUFA6 are both located in the endoplasmic reticulum and mitochondrion. They can encode components of mitochondrial complex I and are involved in mitochondrial respiratory chain complex I assembly. Complex I, composed of 45 subunits, is the first enzyme of the mitochondrial respiratory chain [37]. Mitochondrial complex I and respiration play a critical role in cancer cell proliferation, loss of mitochondrial complex I, and the diminished growth of cancer cells [38]. Mitochondria complex I has shown pro-tumorigenic effects, and it is now a focal point of many targeted therapies using pharmacologic and genetic interventions [39]. In addition, the important role of electron transport chain activity in the targeted therapy of MM has been confirmed in a previous report. Electron transport chain activity is related to venetoclax sensitivity of MM, which is a predictor and target for venetoclax sensitivity in MM [40].

A key regulatory function of COX is to catalyze the transfer of electrons from reduced cytochrome c to oxygen in the mitochondria, which is critical in the oxidative phosphorylation of cells and the process of apoptosis [41]. Mammalian COX is comprised of the coordinated assembly of 13 subunits, in which 3 subunits are encoded by the mitochondrial genomes and 10 subunits are encoded by the nuclear genomes, and their expression varies in different organisms. COX6C and COX5B are encoded by the nuclear genome, then transported to the mitochondria via different pathways, and ultimately integrated into the COX complex. Accumulating evidence demonstrates that COX6C is closely associated with the tumorigenesis and prognosis of breast cancer [42], gastric cancer [43], melanoma [44], and many solid tumors [45]. It is recognized as a valuable biomarker for predicting disease stages and prognosis. Wang et al. also reported that COX6C is a negative prognostic biomarker in MM [46]. Similar to COX6C, COX5B was found to promote cell growth and reduce anticancer drug susceptibility in colorectal cancer cells, which may lead to unfavorable postoperative outcomes for patients with colorectal cancer [47]. Bioenergetic alteration-dependent activation of AMPK was found to occur in hepatoma cells via COX5B regulation of UHMK1 expression [48]. Furthermore, in clear cell renal cell carcinoma, COX5B can also be identified as a prognostic factor [49]. 

USMG5, also known as DAPIT, is located in mitochondria. Overexpression of DAPIT modulates mitochondrial function and alters cellular regulation, promotes anaerobic metabolism, and induces EMT-like transformation in 293T cells. [50]. Further study found that, in various brain regions, pancreas, liver cancers, and other solid tumors and hematological malignancies, DAPIT is among the most duplicated genes. DAPIT overexpression is strongly linked to cancer according to these findings. However, the specific molecular function of USMG5 in hematological malignancies, especially in MM, is still unclear and deserves further study. 

In our study, we also verified that these five genes were significantly related to the stages of MM. According to existing literature, the disease stage is highly individual and is primarily determined by cytogenetic abnormality [3]. Therefore, we assume that the five genes might be directly or indirectly related to high-risk cytogenetic abnormalities. Furthermore, we found there is an association between the high expression of these genes and a poorer prognosis in MM patients from an independent dataset. To further evaluate tumor- and immune-related characteristics, ssGSEA analysis was conducted. The results demonstrated that the immune cell scores differed across samples, reflecting differences in immune function between samples. Subsequently, we examined the correlation between the 5 hub genes and the infiltration of immune cells. According to our findings, there was a negative correlation between the expression of NDUFB8, NDUFA6, COX6C, COX5B, and USMG5 and the infiltration, functions, and pathways of B cells, central memory T cells, and regulatory T cells. In comparison, the expression of these 5 genes exhibited a positive correlation with T cells and T-helper 2 cells. Our results indicate that NDUFB8, NDUFA6, COX6C, COX5B, and USMG5 might influence the immune microenvironment of multiple myeloma patients, as well as the response to treatment and prognosis of patients with this disease. To the best of our knowledge, we pioneered to leverage the genomic resources by integrating single-cell RNA-seq and bulk RNA-seq data in multiple myeloma to provide novel insight into disease progression, and to identify potential biomarkers for precision treatment options. Nonetheless, the present study still has limitations. Further prospective randomized study is required to validate our results and to investigate the relationship between the differentially expressed genes with other known cytogenetic abnormalities. We will also be able to compare more patients within the same treatment regimen in future studies to improve our understanding of how treatments affect tumors. Moreover, further experimental evidence is needed to elucidate the potential mechanism underlying differentially expressed genes in multiple myeloma tumorigenesis and progression.

## 5. Conclusions

In summary, in our study, multiple myeloma progression was characterized by tumor cell heterogeneity. The combined analysis of scRNA-Seq and bulk RNA-Seq may allow us to elucidate molecular events during clonal progression, and further, clarify novel prognostic biomarkers of the initiation and progression of MM. These findings reveal the molecular and cellular complexity of MM progression, and facilitate the design of precise risk stratification and treatment strategies based on novel molecular biomarkers. 

## Data Availability

Not applicable.

## Supplementary Materials

The following supporting information can be downloaded at: https://www.mdpi.com/article/10.3390/biom12121855/s1.
