# Supplementary material for: Integrative Analysis of Bulk RNA-Seq and Single-Cell RNA-Seq Unveils Novel Prognostic Biomarkers in Multiple Myeloma
Source: Biomolecules. 2022 Dec 12;12(12):1855. doi: 10.3390/biom12121855 (PMC9776050; doi:10.3390/biom12121855)
Supplement: Supplementary file 1 [file biomolecules-12-01855-s001.zip › Table S1.pdf]

Primer Sequences

COX6C	Forward Primer	TTCTGGGCTCCCTACAACATT
	Reverse Primer	TTGGTCCAACCTGTTAGAGCTA
COX5B	Forward Primer	ATGGCTTCAAGGTTACTTCGC
	Reverse Primer	CCCTTTGGGGCCAGTACATT
NDUFA6	Forward Primer	GCCCATTTTCAGTCGGGACA
	Reverse Primer	TTGGCGCTTCTGTTTCATGG
NDUFB8	Forward Primer	GTTGAACTGGGGTGAACCGA
	Reverse Primer	CCAAGGCAGAGAGAAAGCCT
USMG5	Forward Primer	GCGGCTTTGGCAGATTGAAA
	Reverse Primer	CTTCTCCAGGCATGGGAACTT
